# Supplementary figures and images for: Paper-Based Probes with Visual Response to Vapors from Nitroaromatic Explosives: Polyfluorenes and Tertiary Amines
Source: Molecules. 2022 May 2;27(9):2900. doi: 10.3390/molecules27092900 (PMC9101589; doi:10.3390/molecules27092900)

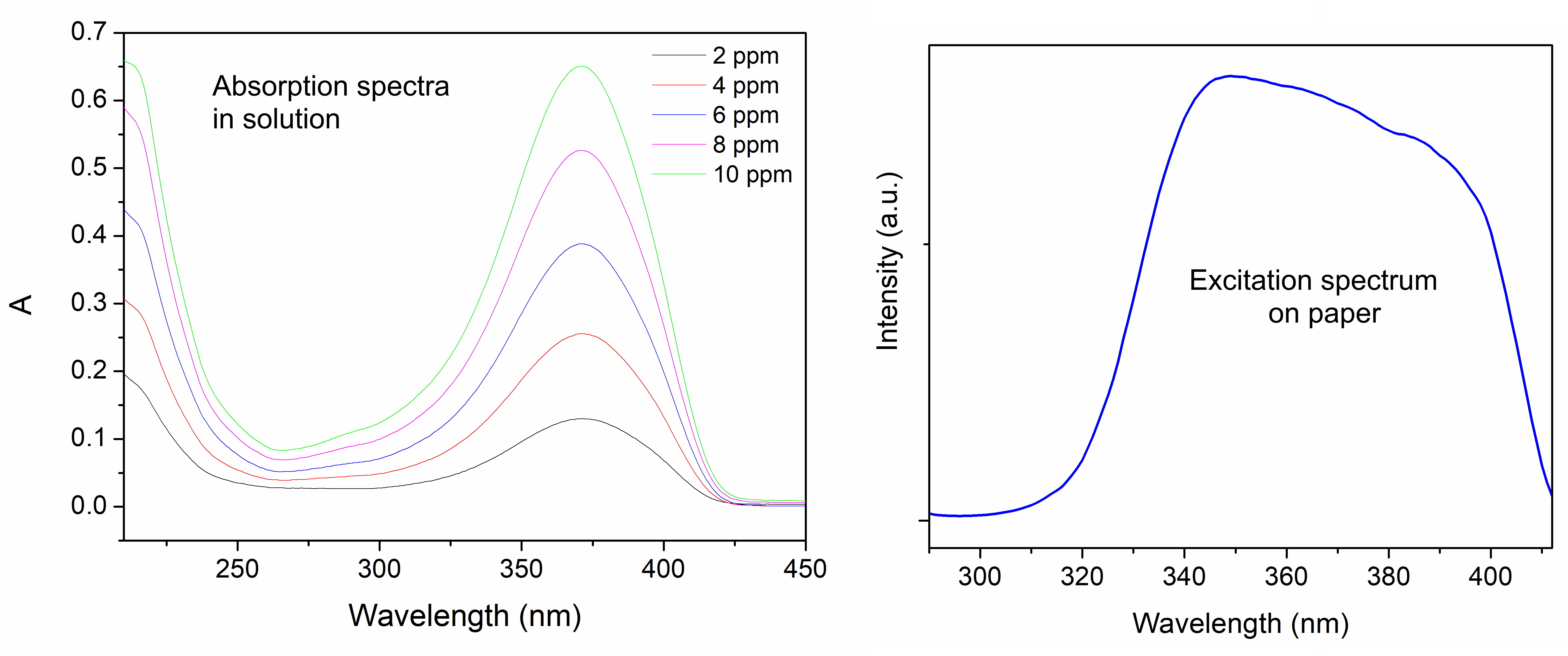

Supplement: Supplementary file 1 [file molecules-27-02900-s001.zip › molecules-1696746-supplementary/Figure S1.jpg]

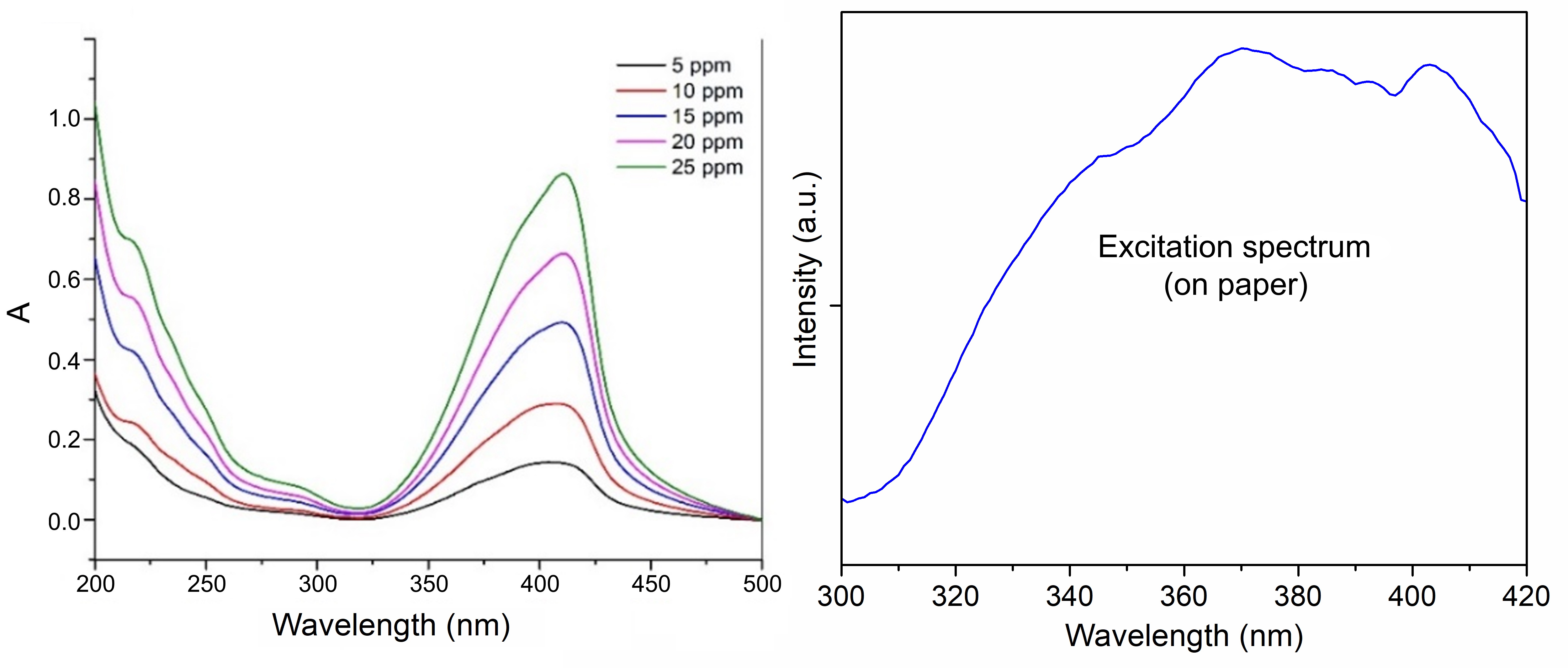

Supplement: Supplementary file 1 [file molecules-27-02900-s001.zip › molecules-1696746-supplementary/Figure S2.jpg]

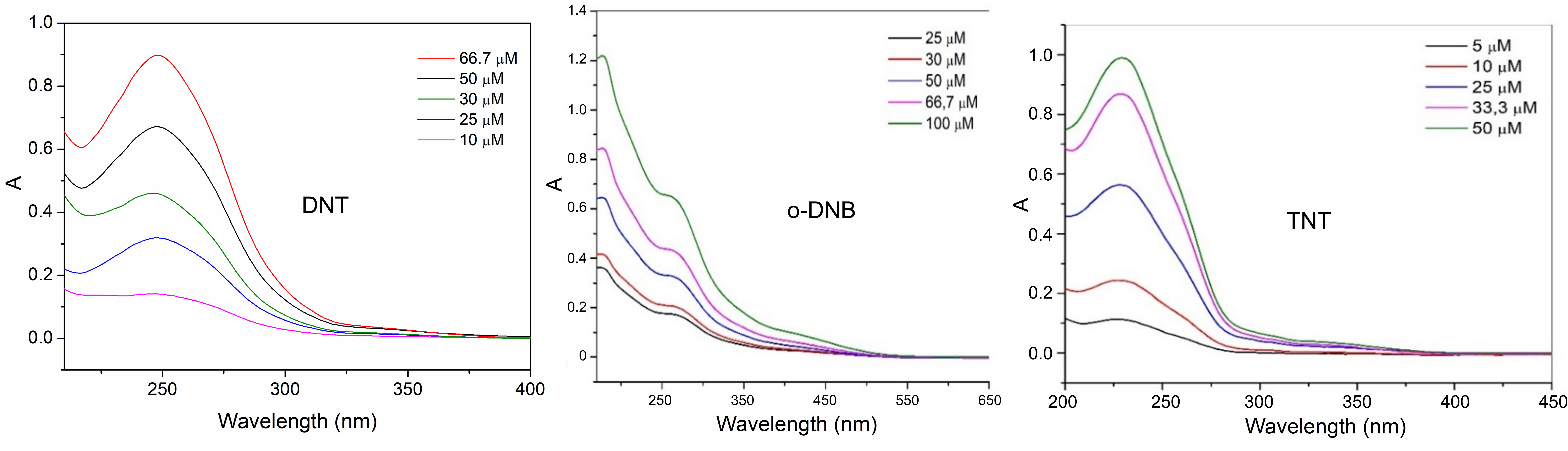

Supplement: Supplementary file 1 [file molecules-27-02900-s001.zip › molecules-1696746-supplementary/Figure S3.jpg]

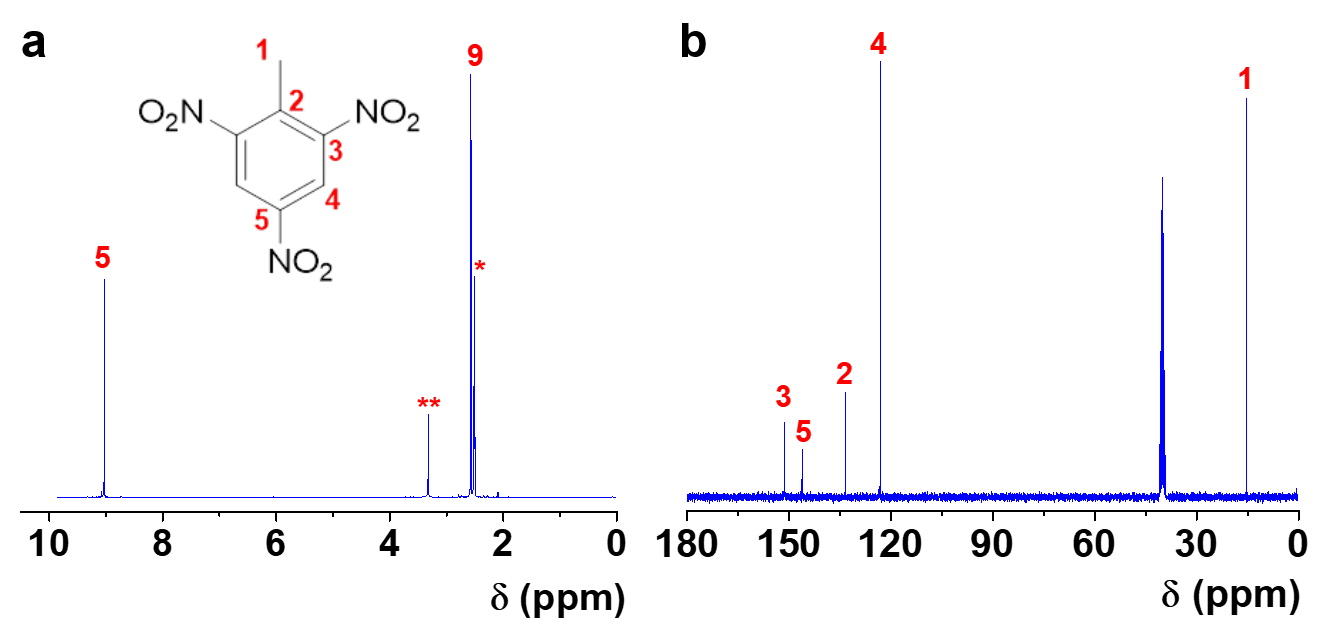

Supplement: Supplementary file 1 [file molecules-27-02900-s001.zip › molecules-1696746-supplementary/Figure S4.jpg]
